# Characterization of Clinically Relevant Strains of Extended-Spectrum β-Lactamase-Producing *Klebsiella pneumoniae* Occurring in Environmental Sources in a Rural Area of China by Using Whole-Genome Sequencing

**DOI:** 10.3389/fmicb.2019.00211

**Published:** 2019-02-12

**Authors:** Xiaohui Chi, Björn Berglund, Huiyun Zou, Beiwen Zheng, Stefan Börjesson, Xiang Ji, Jakob Ottoson, Cecilia Stålsby Lundborg, Xuewen Li, Lennart E. Nilsson

**Affiliations:** ^1^Department of Environment and Health, School of Public Health, Shandong University, Jinan, China; ^2^Department of Clinical and Experimental Medicine, Linköping University, Linköping, Sweden; ^3^State Key Laboratory for Diagnosis and Treatment of Infectious Disease, Collaborative Innovation Center for Diagnosis and Treatment of Infectious Diseases, The First Affiliated Hospital, College of Medicine, Zhejiang University, Hangzhou, China; ^4^Department of Animal Health and Antimicrobial Strategies, National Veterinary Institute, Uppsala, Sweden; ^5^Department of Risk and Benefit Assessment, National Food Agency, Uppsala, Sweden; ^6^Department of Public Health Sciences, Global Health—Health Systems and Policy, Medicines, Focusing Antibiotics, Karolinska Institutet, Stockholm, Sweden

**Keywords:** *Klebsiella pneumoniae*, extended-spectrum β-lactamase, environment, feces, water, multilocus sequence typing, pulsed-field gel electrophoresis, whole-genome sequencing

## Abstract

*Klebsiella pneumoniae* is a gram-negative, opportunistic pathogen, and a common cause of healthcare-associated infections such as pneumonia, septicemia, and urinary tract infection. The purpose of this study was to survey the occurrence of and characterize *K. pneumoniae* in different environmental sources in a rural area of Shandong province, China. Two hundred and thirty-one samples from different environmental sources in 12 villages were screened for extended-spectrum β-lactamase-(ESBL)-producing *K. pneumoniae*, and 14 (6%) samples were positive. All isolates were multidrug-resistant and a few of them belonged to clinically relevant strains which are known to cause hospital outbreaks worldwide. Serotypes, virulence genes, serum survival, and phagocytosis survival were analyzed and the results showed the presence of virulence factors associated with highly virulent clones and a high degree of phagocytosis survivability, indicating the potential virulence of these isolates. These results emphasize the need for further studies designed to elucidate the role of the environment in transmission and dissemination of ESBL-producing *K. pneumoniae* and the potential risk posed to human and environmental health.

## Introduction

The increasing prevalence of antibiotic resistance among bacteria worldwide has become a major issue affecting the global public health (Grundmann, 2014). Extended-spectrum β-lactamases (ESBLs) are a group of enzymes that can hydrolyze penicillins, cephalosporins, and aztreonam, and is one of the main resistance determinants causing resistance to extended-spectrum β-lactam antibiotics in gram-negative bacteria. The main types of ESBLs are SHV, TEM, and CTX-M, the latter of which has become the main epidemic genotype worldwide.

*Klebsiella pneumoniae* is a gram-negative, opportunistic pathogen commonly associated with health-care associated infections (HAIs) such as pneumonia, septicemia, and urinary tract infections (Lee et al., 2012). *K. pneumoniae* has a high capacity to acquire mobile genetic elements such as plasmids containing antibiotic resistance genes (Burmolle et al., 2012). Because of the selection pressure exerted by a widespread use of antibiotics, strains of *K. pneumoniae* endemic at hospitals are often multidrug-resistant (Magiorakos et al., 2012), and treatment of HAIs caused by these strains are often difficult (Hou et al., 2015; Huang et al., 2015). Particularly problematic are strains of *K. pneumoniae* which are resistant to the last-resort antibiotics carbapenems (Little et al., 2014; Tijet et al., 2014; Cubero et al., 2016). For these reasons, monitoring of *K. pneumoniae* in the clinics is essential in the management of HAIs.

In recent years, the environment has been highlighted as an important factor in the dissemination of antibiotic-resistant bacteria and antibiotic resistance genes (Machado and Bordalo, 2014). An important factor in facilitating the dissemination of antibiotic resistance is likely selection pressures exerted by antibiotic residues in waste from anthropogenic wastewater and discharge from animal production facilities. The impact and effect of environmental contamination of antibiotic-resistant bacteria on human and environmental health has not yet been fully elucidated. A first step to determining this is to survey, monitor, and characterize the clinically important bacteria with clinically relevant antibiotic resistance determinants occurring in the environment, however, data on the occurrence and characteristics of ESBL-producing *K. pneumoniae* in environmental sources is scarce. Additionally, the few studies performed usually focus on prevalence in a single environmental matrix such as a body of water or animal manure (Mollenkopf et al., 2013; Zhang et al., 2015), and whole-genome characterization of the isolates are lacking (Zadoks et al., 2011; Ben Said et al., 2015).

In the current study, we screened 231 environmental samples for ESBL-producing *K. pneumoniae* and performed whole-genome sequencing on the 14 positive isolates detected, in order to survey the occurrence of and characteristics of this bacterial pathogen in different environmental sources in a rural area of Shandong province, China.

## Materials and Methods

### Sample Collection

**Figure 1:**
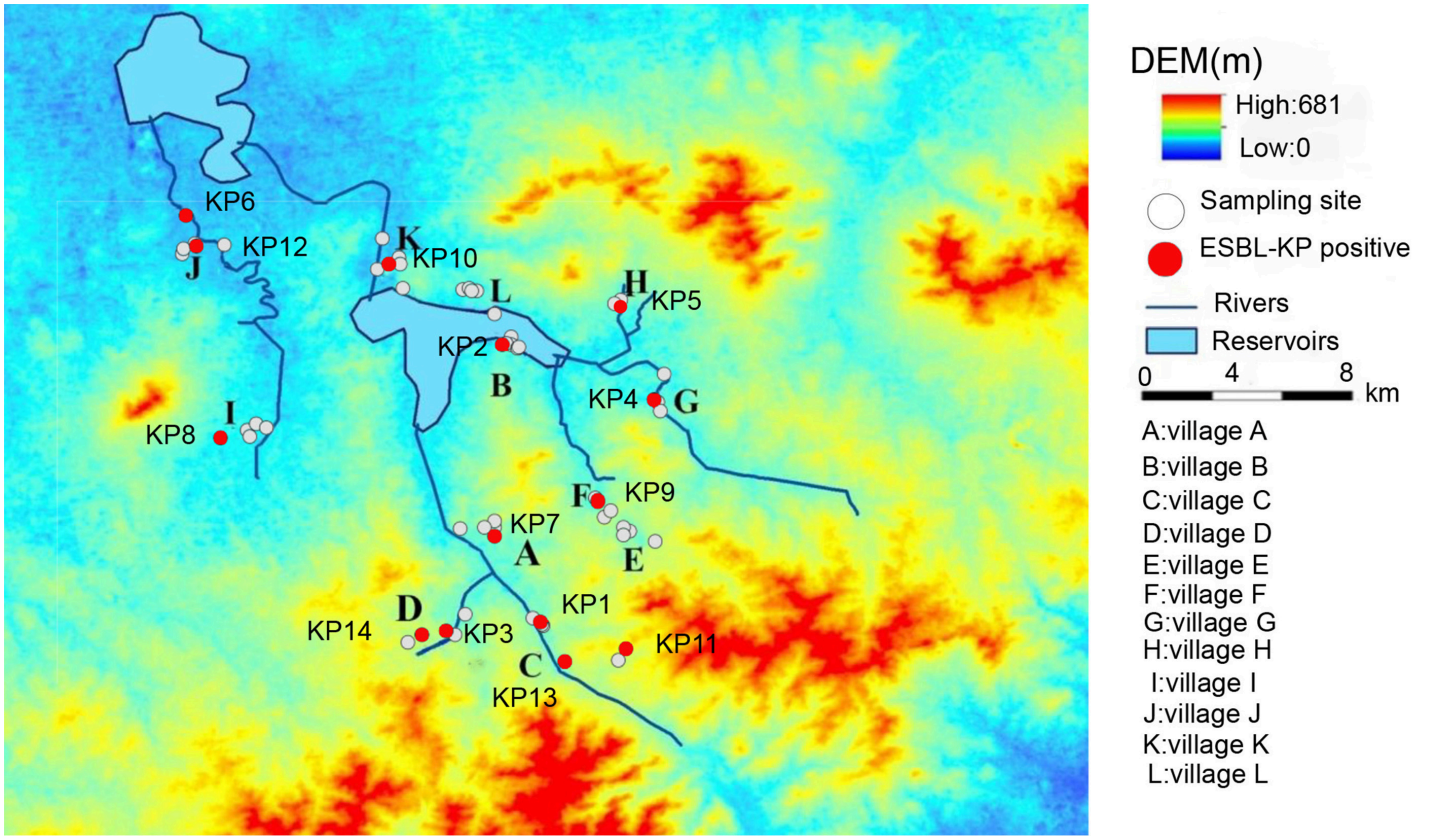
Map showing village locations (A–L) and the surrounding area in a region of rural Shandong, China, where 231 environmental samples were collected for screening for ESBL-producing *K. pneumoniae*. White spots denote locations where samples were negative for ESBL-producing *K. pneumoniae* and red spots denote locations were samples were positive for ESBL-producing *K. pneumoniae*.

Samples were collected in a rural area of Shandong Province, China, in July 2015 (Figure 1). Details of the sampling procedure have been described elsewhere (Sun et al., 2018). Pig manure, wild bird feces, vegetables (cucumber, beans, and chives), and environmental samples were collected with ESwab tubes (Copan, Brescia, Italy) from the study villages. Sample types included: pig manure (*n* = 30), wastewater (*n* = 42), soil (*n* = 23), vegetables (*n* = 23), outlet sediment (*n* = 14), drinking water (*n* = 44), river water (*n* = 25), river sediment (*n* = 24), and feces from wild birds (*n* = 6). All samples were put in cool-boxes with ice-packs (4–8°C) upon collection and were transported for ~6 h to Shandong Center for Disease Control and Prevention (Jinan). Upon arrival, all samples were put in long-term storage at −80°C until cultivation.

### Cultivation of Samples and Screening for ESBL-Producing *K. pneumoniae*

Prior to cultivation, the samples were collected from the low temperature freezers and allowed to reach room temperature. All samples were screened for ESBL-producing *K. pneumoniae* by cultivation on ChromID ESBL agar (bioMérieux, Marcy l'Etoile, France). For water, soil, river sediment, and vegetable samples, a pre-enrichment step in 2–3 ml Brain Heart Infusion Broth (BD, Sparks, USA) was performed overnight before being applied on the screening plates. Each sample was cultivated unselectively on a sheep blood agar plate (Babio, Jinan, China) to verify the viability of bacteria in the sample. All samples were applied on agar plates by a primary streak using the brush in the ESwab and then sub-streaked using a sterile 1 μl plastic loop. The agar plates were incubated for 18–24 h at 37°C. After incubation, growth was recorded and suspected *K. pneumoniae* colonies were identified based on color and morphology, according to the manufacturer's instructions (bioMérieux), sub-cultured on CHROMagar Orientation agar (CHROMagar Company, Paris, France), and incubated overnight at 37°C. From the CHROMagar Orientation agar, suspected *K. pneumoniae* were transferred to storage tubes with glycerol and stored in −80°C until further analyses. After applying samples on the respective agar plates, the remaining fluid in the ESwab tubes were transferred to 2 ml tubes and mixed with 30% glycerol for long-term storage at −80°C.

### Verification of ESBL-Producing *K. pneumoniae*

Species confirmation of presumptive *K. pneumoniae* isolates were performed with matrix-assisted laser desorption ionization time-of-flight mass spectrometry (MALDI-TOF-MS) by using a Shimadzu with saramis premium (Shimadzu Corporation, Kyoto, Japan). ESBL-production was verified by using the double disc diffusion method using cefotaxime, ceftazidime, each alone, and in combination with clavulanic acid according to the manufacturer's protocol (Oxoid, Basingstoke, UK). *Klebsiella pneumoniae* ATCC700603 was used as control strain.

### Antibiotic Susceptibility Testing of ESBL-Producing *K. pneumoniae*

The confirmed ESBL-producing isolates were cultured on blood agar overnight at 37°C and were subsequently tested for susceptibility to 17 different antibiotics listed in Table 1. Antibiotic susceptibility was determined by evaluating minimal inhibitory concentrations (MICs) obtained with the agar dilution method by using the protocol recommended by the Clinical and Laboratory Standards institute (CLSI). Susceptibility was determined in accordance with the European Committee on Antimicrobial Susceptibility Testing, breakpoint tables for interpretation of MICs and zone diameters version 8.1, 2018 (http://www.eucast.org) for all antibiotics except tetracycline and nitrofurantoin, for which CLSI breakpoints version 2017 were used. Since clinical breakpoints of florfenicol are not available for Enterobacteriaceae at EUCAST or CLSI, a resistance breakpoint of >16 mg/L, based on the epidemiological cut-off values for the closely related *Escherichia coli* and *Salmonella* spp. available at EUCAST, was used. Isolates were considered to be multidrug-resistant if resistant to antibiotics from more than two different classes of antibiotics (Magiorakos et al., 2012).

**Table 1:** Antibiotic susceptibility profiles of ESBL-producing *K. pneumoniae* of isolates from river water (RW), rivers sediment (RS), pig manure (PM), soil (S), and vegetables (V).

**Isolate**	**Source**	**MIC (mg/L)**^**a**^																
		**CL**	**AMC**	**TZP**	**CTX**	**CAZ**	**MEM**	**ETP**	**IPM**	**GEN**	**AMK**	**TE**	**TGC**	**CIP**	**SXT**	**FOF**	**F**	**FFC**
KP1	RW	0.125	**16**	**32**	2	2	0.015	0.015	0.25	**32**	4	**128**	2	**16**	**>16**	**256**	**256**	**>32**
KP2	RW	0.06	**16**	**256**	**8**	0.5	0.03	0.06	0.125	**32**	8	**128**	2	**16**	**>16**	**256**	**512**	**>32**
KP3	RW	0.125	**16**	**128**	**8**	**16**	0.015	0.015	0.06	**32**	2	**128**	2	**0.5**	**>16**	**128**	**256**	4
KP4	RW	0.125	**32**	**256**	**32**	1	0.015	0.015	0.125	**128**	2	**64**	2	**8**	**8**	**>256**	**256**	**>32**
KP5	RW	0.125	**32**	**256**	**>32**	1	0.03	0.015	0.125	1	2	**128**	2	**1**	**8**	**>256**	**256**	8
KP6	RW	0.125	**32**	**256**	**>32**	1	0.015	0.03	0.125	**64**	4	**128**	4	**16**	**8**	**256**	**256**	**>32**
KP7	PM	0125	**16**	**256**	**8**	0.5	0.015	0.06	0.125	**32**	4	**128**	2	**16**	**>16**	**256**	**256**	**>32**
KP8	PM	0.125	**16**	4	**8**	1	0.015	0.015	0.125	**16**	2	**128**	1	**4**	**8**	**256**	**128**	**32**
KP9	S	0.125	**32**	**256**	**4**	1	0.015	0.03	0.125	**64**	4	**128**	2	**8**	**8**	**512**	**256**	**>32**
KP10	S	0.06	**32**	**256**	**>32**	1	0.015	0.05	0.125	**64**	2	**128**	4	**0.5**	**8**	**>256**	64	2
KP11	V	0.125	**16**	**256**	**8**	1	0.015	0.015	0.125	**16**	2	**128**	1	**1**	**>16**	**256**	64	**>32**
KP12	V	0.06	**32**	**256**	**>32**	1	0.03	0.03	0.125	**>128**	8	**128**	2	**4**	**8**	**>256**	**256**	**>32**
KP13	RS	0.125	**16**	**256**	**8**	0.5	0.015	0.06	0.125	**32**	4	**128**	0.5	**16**	**>16**	**256**	**128**	**>32**
KP14	WW	0.125	**16**	**256**	**8**	1	0.015	0.03	0.06	**32**	4	**128**	1	**16**	**>16**	**256**	**512**	**>32**

Resistance is indicated in bold and intermediate is indicated in underlined.

a *MICs were determined by agar dilution methods for all antibiotics except for colistin, for which broth microdilution was used. Resistance is indicated in bold. CL, colistin; AMC, amoxicillin/clavulanate; TZP, piperacillin/tazobactam; CTX, cefotaxime; CAZ, ceftazidime; MEM, meropenem; GEN, gentamicin; AMK, amikacin; TE, tetracycline; TGC, tigecycline, CIP, ciprofloxacin; SXT, trimethoprim/sulfamethoxazole; FOF, fosfomycin; F, nitrofurantoin; FFC, florfenicol. Antimicrobial susceptibility profiles were determined using clinical breakpoints recommended by the EUCAST criteria (http://www.eucast.org/clinical_breakpoints/), except for tetracycline and nitrofurantion, for which the Clinical and Laboratory Standards Institute (CLSI, 2017) breakpoints were used, and florfenicol, for which a resistance breakpoint of >16 mg/L, based on the epidemiological cut-off values for the closely related Escherichia coli and Salmonella spp. available at EUCAST, was used*.

### Determination of Clonal Relatedness With Pulsed-Field Gel Electrophoresis

The clonal relatedness of the ESBL-positive *K. pneumoniae* isolates was determined via pulsed-field gel electrophoresis (PFGE) as previously described (Chen et al., 2017). Briefly, DNA plugs were digested using XbaI restriction enzyme (Takara Bio Inc., Kyoto, Japan) for 2 h. PFGE was undertaken on a CHEF-DR III (Bio-Rad, Hercules, CA, USA) using the following parameters: running time 18 h, temperature 14°C, field strength 6 V/cm^2^, angles 120°, initial pulse time 2.2 s, final pulse time 63.8 s. Two isolates were considered related to each other if the homology of the digested patterns was more than or equal to 80%.

### Serum Bactericidal and Phagocytosis Assays

Serum bactericidal and phagocytosis assays were modified from previously described protocols (O'Shaughnessy et al., 2012; Kobayashi et al., 2016). In short, blood was venesected from 10 healthy persons for the serum bactericidal assay and from three healthy persons for the phagocytosis assay. The participants had provided written informed consent prior to participation in the study. Blood was left to clot at 4°C for 8 h, and subsequently centrifuged at 4°C. Inactivated serum was acquired by incubation in a water bath at 56°C for 30 min. Serum bactericidal assays were performed by adding 20 μL of bacterial suspension (10^8^ cfu/mL) to 180 μL of normal or inactivated serum, and incubating the mixture at 37°C for 1 h after which the mixture was quickly placed on ice. From the reaction mixture, 50 μL were diluted 100-fold in PBS and viable count was performed by plating 100 μL on Mueller-Hinton agar and inoculating for 18–24 h. Serum bactericidal assays for each isolate were performed in duplicates at three separate times, and the survival rates were calculated as the ratios between the viable counts in the normal and inactivated serum, based on the means of the replicates.

For the phagocytosis assays; neutrophils were purified from the freshly drawn blood, and enumerated in a Neubauer chamber. Neutrophils were cultured in 24-well plates for 30 min. The phagocytosis assay was performed by adding either 10^6^ neutrophils or an equal volume of autoclaved water (for the control experiments) to a mixture of 200 μL of bacterial suspension (10^8^ cfu/mL), 100 μL of inactivated serum, and 600 μL phosphate-buffered saline, with subsequent incubation of the mixture at 37°C for 1 h, followed by termination of the reaction by adding 100 μL 0.1% Triton X-100 (Shanghai Aladdin Biochemical Technology, Shanghai, China) and placing on ice for 15 min. Bacteria were enumerated by viable count performed by plating 100 μL of the reaction mixture, diluted 1000-fold dilution in PBS, on Mueller-Hinton agar plates, and inoculating for 18–24 h. Phagocytosis assays for each isolate were performed in duplicates at two separate times, and the survival rates were calculated as the ratios between the viable counts in the phagocytosis assay and the controls, based on the means of the replicates.

### Whole-Genome Sequencing of ESBL-Producing *K. pneumoniae*

DNA of all ESBL-positive isolates was extracted from pure cultures of *K. pneumoniae* using a Gentra Puregene Yeast/Bact. Kit (QIAGEN, Hilden, Germany). Whole-genome sequencing was performed on the extracted DNA by Novogene (Beijing, China) using the Illumina HiSeq sequencing platform. Raw sequences were assembled by using SPAdes 3.11 (Anton et al., 2012) (Table S1) and annotated via RAST. All draft genomes were deposited at NCBI (accession number: RCGE00000000-RCGR00000000). Occurrence of antibiotic resistance genes and multilocus sequence typing (MLST) was performed by querying the databases at the Center for Genomic Epidemiology (www.genomicepidemiology.org). Lipopolysaccharide-based serotyping (O-type) and capsule polysaccharide-based serotyping (K-type) were performed by using Kaptive Web (Wick et al., 2018). Presence of virulence genes were determined by using the Institut Pasteur MLST and whole genome MLST databases (http://bigsdb.pasteur.fr/klebsiella/klebsiella.html). In addition, the operons encoding type 1 fimbriae (*fimABCDEFGHIK*) and type 3 fimbriae (*mrkABCDFHIJ*) were evaluated, as were the gene sequences for the outer membrane porins (OMPs) OmpK35 and OmpK36. *ompK36* allele group was determined based on genetic polymorphisms between bp 500 and 1,000, as previously described (Du et al., 2018). The genetic environment surrounding the CTX-M-type ESBL-genes were annotated and investigated using Easyfig 2.2.3. All genomes in the study dataset were annotated using the Prokka prokaryotic (Seemann, 2014) annotation pipeline, and using Roary: the Pan Genome Pipeline (Page et al., 2015) to obtain a system of linked qualified single nucleotide polymorphisms (SNPs) identified by mapping raw reads to core genes development.

### Statistical Analysis

Mann-Whitney tests were used to determine differences in MICs for cefoxitin and ceftazidime between isolates carrying genes encoding CTX-M ESBLs of different groups. Viable counts from the serum bactericidal and phagocytosis assays were compared with their respective controls and differences were determined using repeated measures ANOVA with Bonferroni multiple comparison tests performed *post-hoc*. All statistical tests were carried out using Prism 5 for Windows.

## Results

### Screening for ESBL-Producing *K. pneumoniae*

A total of 14 (6%) isolates of ESBL-producing *K. pneumoniae* were isolated from 231 environmental samples. The positive rate of ESBL-producing *K. pneumoniae* isolates in different environmental matrices were: river water 24% (6/25), soil 9% (2/23), vegetables 9% (2/23), pig manure 7% (2/30), river sediment 4% (1/24), and wastewater 2% (1/42). No isolates were detected in the sewage outlet sediment, drinking water, or wild bird fecal samples.

### Antibiotic Susceptibility Testing

The antibiotic susceptibility testing (Table 1) showed that all isolates were susceptible to amikacin, colistin and the carbapenems meropenem, imipenem, and ertapenem. Susceptibility to ceftazidime was high (86%). None of the isolates were susceptible to cefotaxime, amoxicillin-clavulanic acid, ciprofloxacin, trimethoprim-sulfamethoxazole, tetracycline, nitrofurantoin or fosfomycin. Susceptibility rates to gentamicin (7%), piperacillin-tazobactam (7%), and florfenicol (21%) were low. The isolates showed a low (29%) susceptibility rate to tigecycline. All isolates were multidrug-resistant (i.e., resistant to antibiotics of more than two classes of antibiotics).

### Antibiotic Resistance Genes

**Figure 2:**
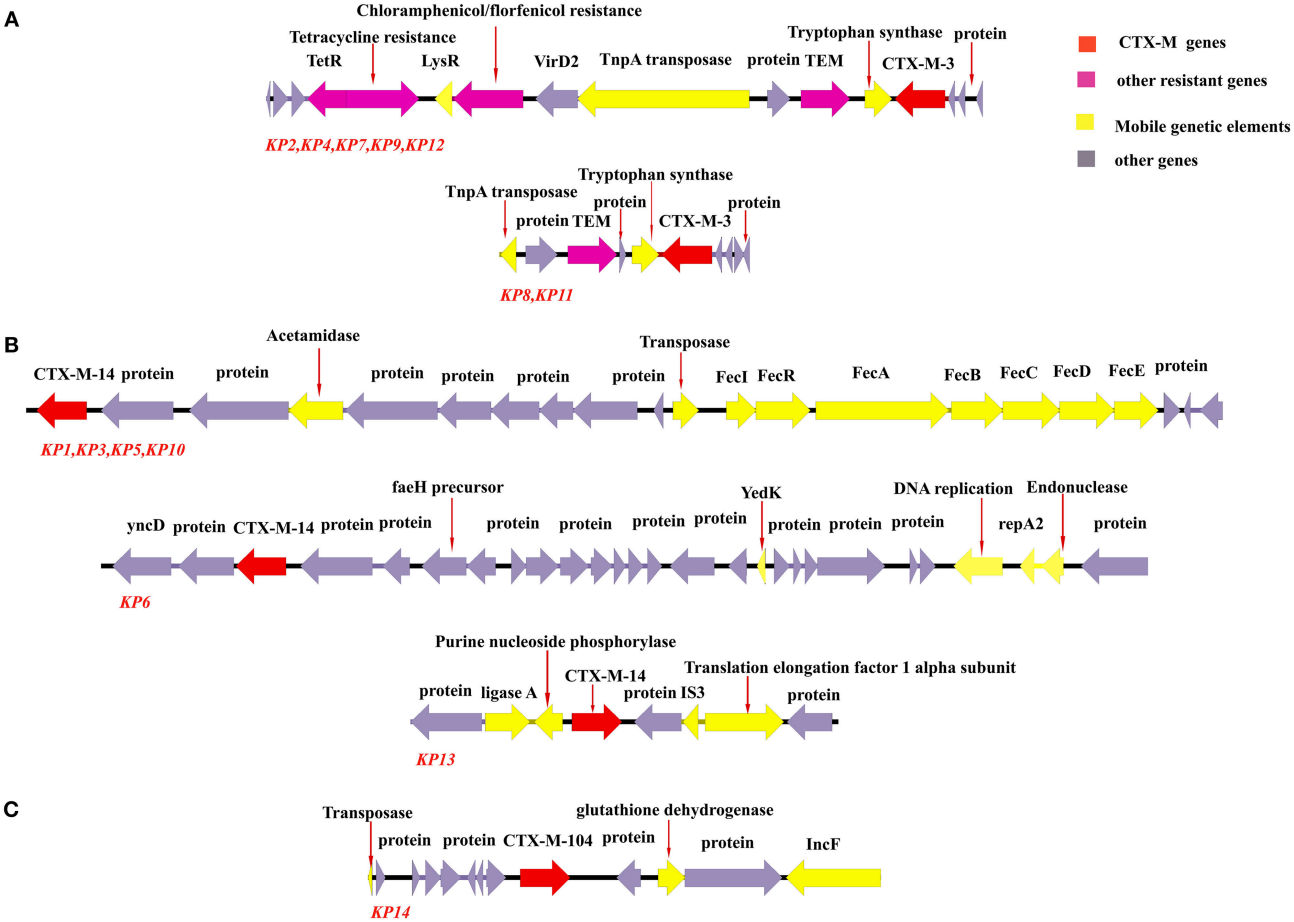
Genetic environment of CTX-M genes among *K. pneumoniae* isolated from environmental sources. Arrows represent direction of transcription. Red open reading frames (ORFs) denote the CTX-M genes, purple ORFs denote other antibiotic resistance genes, yellow ORFs denote genes related to mobile genetic elements, and gray ORFs denote other genes or genes of unknown function. **(A)** The genetic environments of *bla*_CTX−M−3_ were similar in isolates KP2, KP4, KP7, KP9, and KP12. The two isolates KP8 and KP11 also shared a similar genetic environment surrounding *bla*_CTX−M−3_. **(B)** Isolates KP1, KP3, KP5, and KP10 all carried *bla*_CTX−M−14_ and shared the same genetic environment surrounding the gene, whereas isolates KP6 and KP13 had unique genetic environments surrounding *bla*_CTX−M−14_. **(C)** The genetic environment of *bla*_CTX−M−104_ in the one isolate in the study which carried the gene.

Data on antibiotic resistance genes are presented in Table 2. All isolates carried CTX-M-type ESBL-genes. Seven isolates (50%) carried *bla*_CTX−M−3_, six isolates (43%) carried *bla*_CTX−M−14_, and only one isolate carried *bla*_CTX−M−104_. Additionally, the ESBL-gene *bla*_SHV−27_ was detected in two isolates. The genetic environment surrounding the CTX-M genes are shown in Figure 2. Two distinct genetic environments and three distinct genetic environments were determined among the isolates carrying *bla*_CTX−M−3_ and *bla*_CTX−M−14_, respectively.

**Table 2:** Antibiotic resistance genes and STs of ESBL-producing *K. pneumoniae* isolates from environmental samples.

**Isolate**	**Source**	**Antibiotic resistance genes**	**MLST**
KP1	RW	ARR-3*, strA, strB, aph(3′)-*Ia*, aadA16, aac(3)-*IId*, qnrS1, oqxA, oqxB, qnrB52, fosA, mph(A), sul1, sul2, dfrA27, tet(A), floR, aac(6′)*Ib-cr*, bla*_TEM−1B_*, bla*_SHV−27_*, bla*_CTX−M−14_	ST967
KP2	RW	ARR-3*, strA, strB, aph(3′)-*Ia*, aadA16, aac(3)-*IId*, qnrS1, oqxA, oqxB, qnrB49, fosA, mph(A), sul1, sul2, dfrA27, tet(A), floR, aac(6′)*Ib-cr*, bla*_CTX−M−3_*, bla_*S*_*_HV−28_*, bla*_TEM−1B_	ST15
KP3	RW	*fosA, qnrS1, oqxA, oqxB, aac(3)-*IId*, strA, strB, sul1, sul2, dfrA1, tet(A), bla_*T*_*_EM−1B_*, bla*_CTX−M−14_*, bla_*S*_*_HV−1_	ST101
KP4	RW	ARR-3*, strA, strB, aph(3′)-*Ia*, aadA16, aac(3)-*IId*, qnrS1, oqxA, oqxB, fosA, mph(A), sul2, sul1, dfrA27, tet(A), floR, aac(6′)*Ib-cr*, bla*_CTX−M−3_*, bla*_SHV−1_*, bla_*T*_*_EM−1B_	ST3003
KP5	RW	*fosA, qnrS1, oqxA, oqxB, sul1, dfrA1, tet(A), bla*_SHV−11_*, bla*_CTX−M−14_	ST659
KP6	RW	ARR-3*, strA, strB, aph(3′)-Ia, aadA16, aac(3)-IId, sul1, sul2, fosA, mph(A), qnrS1, oqxB, oqxA, qnrB49, dfrA27, tet(A), floR, aac(6′)*Ib-cr*, bla*_SHV−1_*, bla*_CTX−M−14_	ST314
KP7	PM	ARR-3*, strA, strB, aph(3′)-*Ia*, aadA16, aac(3)-*IId*, oqxA, oqxB, qnrS1, qnrB49, fosA, mph(A), sul1, sul2, dfrA27, tet(A), floR, aac(6′)*Ib-cr*, bla*_CTX−M−3_*, bla*_SHV−26_*, bla*_TEM−1B_	ST1967
KP8	PM	ARR-3*, fosA, qnrS1, qnrB49, oqxA, oqxB, strA, strB, aph(3′)-*Ia*, aadA16, aac(3)-*IId*, mph(A), sul1, sul2, dfrA27, tet(A), floR, aac(6′)*Ib-cr*, bla*_TEM−1B_*, bla*_SHV−11_*, bla*_CTX−M−3_	New
KP9	S	ARR-3*, strA, strB, aph(3′)-*Ia*, aadA16, aac(3)-*IId*, qnrS1, oqxA, oqxB, fosA, mph(A), sul2, sul1, dfrA27, tet(A), floR, aac(6′)*Ib-cr*, bla*_CTX−M−3_*, bla*_SHV−1_*, bla*_TEM−1B_	ST999
KP10	S	*fosA, sul1, aac(3)-*IId*, oqxA, oqxB, qnrS1, dfrA1, tet(A), bla*_CTX−M−14_, *bla*_SHV−82_	ST1738
KP11	V	ARR-3*, fosA, oqxA, oqxB, qnrB49, strA, strB, aph(3′)-*Ia*, aadA16, aac(3)-*IId*, mph(A), sul1, sul2, dfrA27, tet(A), floR, aac(6′*)Ib-cr*, bla*_TEM−1B_, *bla*_SHV−11_*, bla*_CTX−M−3_	New
KP12	V	ARR-3*, fosA, oqxA, oqxB, strA, strB, aph(3′)-*Ia*, aadA16, aac(3)-*IId*, mph(A), sul2, sul1, dfrA27, tet(A), floR, aac(6′)*Ib-cr*, bla*_CTX−M−3_*, bla*_SHV−27_*, bla*_TEM−1B_	ST661
KP13	RS	ARR-3*, fosA, oqxA, oqxB, aph(3′)-*Ia*, aadA16, mph(A), sul1, dfrA27, tet(D), floR, catA2, aac(6′)*Ib-cr*, bla*_SHV−11_*, bla*_CTX−M−14_	ST11
KP14	WW	ARR-3*, strB, aadA16, aadA2, aac(3)-*IId*, aph(3′)-*Ia*, qnrB6, oqxA, oqxB, fosA, erm(B), sul1, sul2, dfrA27, tet(A), tet(D), floR, aac(6′)*Ib-cr*, cfr, bla*_SHV−11_*, bla*_TEM−1A_*, bla*_CTX−M−104_	ST258

*The samples were collected from river water (RW), pig manure (PM), soil (S), vegetable (V), river sediment (RS), and wastewater (WW)*.

All isolates carried *fosA, oqx*- *sul*-, *dfr*-, and *tet*-genes, encoding fosfomycin-, quinolone-, sulphonamide-, trimethoprim-, and tetracycline resistance, respectively. Aminoglycosides resistance genes were also common (93% of isolates), as were the florfenicol resistance gene *floR* (86% of isolates).

### Serotyping and Virulence-Associated Genes

Serotyping was performed based on both lipopolysaccharide (O-serotype) and capsular polysaccharide (K-serotype) (Table 3). The O-serotypes were distributed as follows; O2 (*n* = 5), O1 (*n* = 4), O3 (*n* = 2), OL101 (*n* = 1), and OL103 (*n* = 1). One isolate (KP4) had poor match confidence against any known O-type (<90% coverage and two truncated/missing genes) and was determined to be non-typeable. The K-serotype-associated K loci among the isolates were diverse; two isolates had KL34, whereas KL3, KL18, KL23, KL28, KL31, KL38, KL49, KL57, KL106, KL107, and KL127 were found in one isolate each. One isolate (KP4) had poor match confidence against any known K-serotype and was determined to be non-typeable.

**Table 3:** Serotypes, virulence characteristics, and virulence-associated genes of ESBL-producing *K. pneumoniae* from environmental samples.

	**KP1**	**KP2**	**KP3**	**KP4**	**KP5**	**KP6**	**KP7**	**KP8**	**KP9**	**KP10**	**KP11**	**KP12**	**KP13**	**KP14**
MLST	ST967	ST15	ST101	ST3003	ST659	ST314	ST1967	New	ST999	ST1738	New	ST661	ST11	ST258
O serotype	O1	OL103	O1	N.D.	O2	O2	O2	O1	OL101	O3	O1	O2	O3	O2
K locus	KL18	KL49	KL106	N.D.	KL3	KL23	KL57	KL34	KL127	KL38	KL34	KL28	KL31	KL107
Serum survival	74%	2%	82%	34%	51%	30%	53%	111%	47%	1%	122%	68%	67%	39%
P value	<0.05	<0.001		<0.001	<0.001	<0.001	<0.001		<0.001	<0.001			<0.05	<0.01
Phagocytosis survival	74%	104%	94%	75%	75%	78%	81%	104%	67%	74%	55%	97%	108%	75%
P value											<0.01			
OmpK35	WT	WT	WT	WT	WT	WT	WT	WT	WT	WT	WT	WT	Q68Stop	WT
*ompK36* allele group	C	D	C	A	D	D	N.D.	A	D	D	A	A	A	A
Type 1 fimbriae	+	+	+	+	+	+	+	+	+	+	+	+	+	+
Type 3 fimbriae	+	+	+	+	+	+	+	+	+	+	+	+	+	+
*kfu*	–	+	+	–	–	–	–	+	–	–	+	–	–	–
*kvg*	–	–	–	–	–	–	–	+	–	–	+	–	–	–
Yersiniabactin	–	–	+	–	–	–	–	–	–	+	–	–	–	–

*P-values are indicated for tests comparing viable counts for serum bactericidal and phagocytosis assays to their respective controls. P > 0.05 are omitted for clarity. WT, wild type; N.D., non-determinable*.

Querying of virulence-associated genes and serotyping was performed *in silico* by using the whole-genome sequencing data (Table 3). All isolates carried the *fim* and *mrk* operons which encode type 1 and type 3 fimbriae, respectively. The *kfu* and the *kvg* operons were detected in four isolates and two isolates, respectively. Additionally, two isolates carried the operon encoding the yersiniabactin siderophore (*ybtSXQPA-irp2-irp1-ybtUTE-fyuA*) (Table 3). The genes encoding the OMPs OmpK35 and OmpK36 were also evaluated in all isolates. None of the isolates carried an *ompK35* with any mutations clearly affecting the protein function except isolate K13, which carried an *ompK35* with a nucleotide substitution (c202t) engendering a premature stop codon (Q68Stop) causing a 81% truncation of the protein (shortening it from 359 to 67 aa). Based on *ompK36* polymorphisms, the isolates could be divided in three different allele groups; group A (*n* = 7), group D (*n* = 5), and group C (*n* = 2).

### Serum Bactericidal and Phagocytosis Assays

The survivability of the isolates in serum bactericidal and phagocytosis assays were evaluated as the differences in viable counts between the assay and control experiments (Table 3). For the serum bactericidal assay, 10 isolates (71%) had significantly lower survivability compared to the controls (*P* < 0.05); eight of these isolates had survival rates ranging from 30 to 74%. Two isolates (KP2 and KP10) had survival rates of 1 and 2%, respectively, which was significantly lower compared to the other isolates (*P* < 0.01). Four isolates (KP3, KP8, KP11, and KP12) did not differ significantly from the controls and had survival rates ranging from 68 to 122%. In the phagocytosis assay, only one isolate (KP11) had a significantly lower survival rate (55%) compared to the control (*P* < 0.01); the remaining isolates showed survival rates ranging from 67 to 108%.

### Phylogenetic Analysis

**Figure 3:**
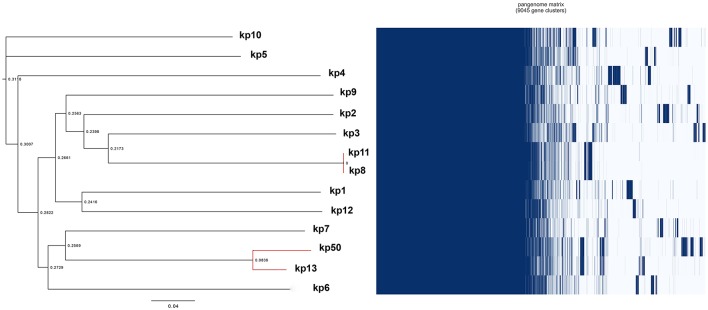
Phylogeny of ESBL-producing *K. pneumoniae* isolated from environmental sources based on differences in SNPs. Red branches indicate isolates which were found related in the PFGE-analysis. The value represents the proportion of the scale of SNP differences.

A total of 13 different STs were found among the 14 ESBL-producing *K. pneumoniae* isolates in this study (Table 2), including ST11, ST15, ST101, ST258, ST659, ST661, ST967, ST999, ST11967, ST3003, and ST1738. Two isolates shared 100% nucleotide sequences (KP8, isolated from vegetables, and KP11, isolated from pig manure) in the MLST genes, but were of a previously undescribed type. These two isolates also presented high similarity in the PFGE-analysis (Figure S1). In addition, the results of the PFGE indicated a close genetic relationship between three other isolates, KP9 (from soil), KP13 (from river sediment), and KP14 (from wastewater). A phylogenetic tree based on SNPs was computed from 9,045 gene clusters obtained from the 14 isolates (Figure 3). The analysis revealed a close genetic relationship between isolates KP8 and KP11, among which no SNP-differences could be discerned. Isolates KP13 and KP14 also showed high genetic similarity.

## Discussion

In the current study, 231 environmental samples were collected from a rural farming area in Shandong province, China, and screened for ESBL-producing *K. pneumoniae*. The results showed that the positive rate among the samples was 6%. Among the different environmental sources sampled, the detection rate was highest in river water (24%), followed by soil (9%), vegetables (9%), pig manure (7%), river sediment (4%), and wastewater (2%). The prevalence in river water was higher than the prevalence in the wastewaters connected to pig discharge. This could reflect the comparatively lower prevalence in pig manure (7%). The river water on the other hand, was mainly influenced by anthropogenic discharge originating from the surrounding community and directly from inhabitants using the water. The higher prevalence in anthropogenically affected water compared to pig discharge wastewater may reflect that human rather than animal sources are more important for the environmental presence of ESBL-producing *K. pneumoniae* in the local area. In China, ESBL-producing *K. pneumoniae* has been reported in spring water adjacent to restaurants and hotels at a mountain resort close to Tai'an, Shandong province (Li et al., 2015) and in water from the Pearl River in Guangzhou (Ye et al., 2017). A fairly high prevalence rate of 36% was also reported in the Yangtze River in Chongqing (Chen et al., 2010). Overall, the findings in the different environmental sources in the study area indicate a need to clarify the role and importance of the environment in the potential for transmission and dissemination of ESBL-producing *K. pneumoniae* and subsequent risk for transmission.

All isolates were susceptible to colistin and carbapenems. The susceptible rate to ceftazidime was high (86%); only one isolate was resistant. Resistance to ceftazidime in *K. pneumoniae* has been shown to be engendered by loss of the OMP OmpK35 (Etsuko et al., 2016). The single resistant isolate in the current study (KP3) carried an *ompK35* with a nonsense mutation which translated into an OmpK35 truncated by 81%, likely rendering the protein nonfunctional. For the antibiotics cefotaxime, amoxicillin-clavulanic acid, piperacillin-tazobactam, ciprofloxacin, trimethoprim-sulfamethoxazole, tetracycline, fosfomycin, gentamicin, and nitrofurantoin, susceptible rates were low, between 0 and 7%. The high MIC-values to these antibiotics correlated to a correspondingly high carriage rate of antibiotic resistance genes. All isolates carried genes conferring resistance to trimethoprim, sulphonamides, quinolones, tetracycline, fosfomycin, and aminoglycosides. The susceptible rate to florfenicol was low (21%), and all of the non-susceptible isolates carried the florfenicol resistance gene *floR*. Additionally, MICs were high for tigecycline, and only a few isolates were susceptible (29%). Notably, all isolates collected in this study were multidrug-resistant.

CTX-M-genes were carried by all isolates in this study. The most prevalent was *bla*_CTX−M−3_, a gene of the CTX-M-1-group, which was detected in seven isolates (50%), followed by *bla*_CTX−M−14_, a gene of the CTX-M-9-group, which was detected in five isolates (43%). MICs of the tested cephalosporins ceftazidime and cefoxitin were not significantly different for isolates with CTX-M-1 group enzymes as compared to isolates with CTX-M-9 group enzymes. CTX-M-genes are the most common genes encoding ESBLs worldwide, and are prevalent among Enterobacteriaceae (Naseer and Sundsfjord, 2011; Bevan et al., 2017). Regarding the epidemiology of CTX-M-genes, most studies performed have focused on *E. coli*. Although there are regional differences pertaining to the distribution of specific CTX-M-genes, *bla*_CTX−M−14_ and *bla*_CTX−M−15_ are predominant in clinical isolates worldwide, which also holds true in China (Shin et al., 2011; An et al., 2012; Xia et al., 2014; Bevan et al., 2017). For *K. pneumoniae* in China, the predominance of certain CTX-M-types differ depending on study. For instance, one study on isolates collected from seven hospital in Beijing reported a predominance of CTX-M-14 (An et al., 2012), whereas another study from a single hospital in Beijing reported CTX-M-10 to be the most common (Li et al., 2012). Another study in a hospital in Guangdong reported CTX-M-9 to be the most common CTX-M-type (Du et al., 2014) The most common gene in the current study, *bla*_CTX−M−3_, is rarely reported in China, although this is likely due to a larger volume of data being available on ESBLs among *E. coli* as compared to *K. pneumoniae*. For instance, a study on clinical isolates of *K. pneumoniae* from hospitals in Jilin, China, showed that 48% of CTM-X-producing isolates carried *bla*_CTX−M−3_ (Li et al., 2014) and another study on *K. pneumoniae* isolates collected at a hospital in Henan, China, showed that *bla*_CTX−M−3_ was carried by 38% of the CTX-M-producers, which was second in prevalence only by carriers of *bla*_CTX−M−14_ (40%) (Guo et al., 2017).

Additionally, the genetic environments surrounding the CTX-M-genes were analyzed (Figure 2). *bla*_CTX−M−3_ (*n* = 7) was located on two distinct genetic fragments among the isolates, whereas *bla*_CTX−M−14_ (*n* = 6) was located on three distinct genetic fragments. These results show that several mobile genetic elements are likely responsible for the dissemination of CTX-M-genes in the local environment. Notably, five isolates (KP2, KP4, KP7, KP9, and KP12) sharing the same genetic environment surrounding *bla*_CTX−M−3_, also carried the florfenicol resistance gene *floR* on the same genetic element, indicating the potential of co-selection of ESBL-producing *K. pneumoniae* via florfenicol exposure. The high variety of ESBL-genes and other antibiotic resistance genes among *K. pneumoniae* isolated from environmental sources indicate the potential for transfer of these genes to other bacteria present in the same sources. Reversely, the *K. pneumoniae* isolates may potentially acquire additional antibiotic resistance determinants from environmentally occurring bacteria.

Isolates of *K. pneumoniae* expressing ESBLs have been linked to higher virulence compared to *K. pneumoniae* isolates lacking ESBLs. In one study, isolates collected from hospital-acquired bacteremia showed that ESBL-producing *K. pneumoniae* were significantly more resistant to serum killing compared to isolates lacking ESBLs (Lin et al., 2016). Another study on isolates from clinical specimens from hospitalized patients found that ESBL-producing *K. pneumoniae* to a higher degree carried both type 1 and type 3 fimbriae and were more capable of invasion of ileocecal and bladder epithelial cells (Sahly et al., 2008). ESBL-production has also been strongly linked to human isolates of *K. pneumoniae*, and it has further been indicated that community-acquired *K. pneumoniae* tend to carry fewer acquired antibiotic resistance genes (Holt et al., 2015). In general, the difference between clinical and environmental isolates of *K. pneumoniae* is not as well-studied.

In the current study, virulence among the environmental isolates of *K. pneumoniae* was evaluated based on serum bactericidal and phagocytosis assays, serotypes, and virulence-associated genes. Ten isolates had significantly lower survivability in serum compared to the controls (*P* < 0.05), two of which (KP2 and KP10) had considerably lower survival rates (1 and 2%, respectively) compared to the other isolates (*P* < 0.01); indicating a difference in degree of survivability in serum for the isolates in this study. Four isolates, KP3, KP8, KP11, and KP12, did not significantly differ in survivability compared to the controls, and had survival rates ranging from 68 to 122%, indicating that these were potentially serum-resistant. For the phagocytosis assay, only one isolate (KP11) had significantly lower survivability compared to the control, implying a high degree of phagocytosis resistance among the isolates. Notably, three isolates (KP3, KP8, and KP12) had high survival rates in both the serum bactericidal and phagocytosis assay, indicating the potential virulence of these isolates.

Serotyping was performed *in silico* by using the whole-genome sequencing data (Table 3). O-serotypes O1, O2, and O3 are the serotypes most commonly isolated from human hosts, and O1 in particular is the most common serotype associated with disease in humans (Rainer et al., 2016) Interestingly, these serotypes predominated among the environmental isolates collected in this study; five isolates were O2, four isolates were O1 and two isolates were O3. Among K-serotypes, K1 and K2 are associated to hypervirulence in *K. pneumoniae* (Rainer et al., 2016), however, none of the isolates in this study carried the KL1 or KL2 loci associated to either of these K-serotypes.

Virulence gene analysis revealed that all isolates carried the *fim* and *mrk* operons which encodes type 1 and type 3 fimbriae, virulence factors ubiquitous in *K. pneumoniae* (Wu et al., 2012; Paczosa and Mecsas, 2016). Both fimbriae mediate adhesion and biofilm formation. Type 1 fimbriae are expressed in the urinary bladder and have been shown to contribute to urinary tract infections (Struve et al., 2009; Paczosa and Mecsas, 2016). Type 3 fimbriae mediate biofilm formation and are involved in adhesion to medical devices, and may be an important factor for *K. pneumoniae* in biofilm-associated infections as well as gaining entry into the host and persistence in the clinical environment rather than facilitating infections in the host (Huang et al., 2009; Struve et al., 2009).

The OMP OmpK36 appears to play a role in *K. pneumoniae* for both antibiotic resistance and virulence, as deficiencies in the protein has been shown to lead to elevated MICs for some antibiotics (likely due to decreased membrane permeability) and lowered virulence (Paczosa and Mecsas, 2016). Genetic polymorphisms in *ompK36* can be used as a basis for classification into four different *ompK36* alleles, each of which is associated with altered protein function. Interestingly, isolates carrying a group C allele have been associated with STs, K-serotypes, and virulence genes associated with hypervirulent *K. pneumoniae* lineages, and have additionally been shown to display higher virulence in a mouse lethality model (Yan et al., 2015; Du et al., 2018). In the current study, two isolates (KP1 and KP3) were found to carry group C alleles of *ompK36*.

Four isolates carried the *kfu* operon, which encodes components of an ABC transport system which mediates uptake of ferric iron. Kfu is associated with hypervirulent strains of *K. pneumoniae*, and its expression has been shown to be important for virulence in mouse models (Luo et al., 2014; Paczosa and Mecsas, 2016). In the current study, two isolates (KP3 and KP10) containing the yersiniabactin virulence operon were found, and where from river water and soil, respectively. Yersiniabactin is a siderophore which helps *K. pneumoniae* survive during respiratory tract infections (Bachman et al., 2011), and has been shown to be strongly associated with infection in humans (Holt et al., 2015). Yersiniabactin is also overrepresented among hypervirulent strains (Paczosa and Mecsas, 2016). One of these isolates (KP3) also carried the *kfu* operon.

The genetic relationship of the isolates was analyzed using MLST, PFGE, and SNPs, and the isolates were found to exhibit a high diversity. For the 14 isolates, 13 different STs were determined (Figure 2, Table 2). The two isolates (KP8, KP11) which shared ST (i.e., identical sequence in MLST genes) belonged to a previously undescribed ST, and showed a close relation in the PFGE analysis. This was confirmed by the SNP-analysis, in which the two isolates had identical SNPs (Figure 3). These isolates also shared identical genetic environments surrounding *bla*_CTX−M−3_ (Figure 2). Other STs detected in this study included clinically relevant STs such as ST11, ST15, ST101, and ST258. ST11 and ST258, both members of clonal group (CG) 258, are the most common carbapenem-resistant *K. pneumoniae* isolated from clinical samples worldwide (Lee et al., 2016). ST258 is commonly reported to cause hospital outbreaks in North America, Latin America, and Europe (Ruiz-Garbajosa et al., 2013; Geraci et al., 2015; Gomez et al., 2016; Lee et al., 2016). ST11 is more commonly reported from hospital outbreaks in Asian countries, including China (Qi et al., 2011; Lee et al., 2016). Recently, a clone of carbapenem-resistant ST11 *K. pneumoniae* having acquired a hypervirulence-plasmid was reported to cause an outbreak at a hospital in Hangzhou, China (Gu et al., 2018). ST15 and ST101 are two other globally occurring strains of *K. pneumoniae* associated with hospital outbreaks. For example, ESBL-producing ST15 has been reported in hospital outbreaks in the Netherlands (Zhou et al., 2016) and Hungary (Damjanova et al., 2008), and carbapenem-resistant ST15 has been reported in outbreaks in Bulgaria (Markovska et al., 2015), Portugal (Vubil et al., 2017), Vietnam (Tada et al., 2017), and Nepal (Chung The et al., 2015). For ST101, hospital outbreaks of carbapenem-resistant *K. pneumoniae* have been reported in Algeria (Loucif et al., 2016) and Greece (Avgoulea et al., 2018). A study in Turkey (Can et al., 2018) on colistin-resistant *K. pneumoniae* isolated from patients found that colistin-resistant ST101 were prevalently carbapenem-resistant, and occurrence of ST101 in the patient was found to be a significant independent predictor of patient mortality. The ST101 isolate in the current study (KP3) had several factors associated with virulence and high-risk clones; it belonged to the commonly disease-associated O-serotype O1, carried a group C *ompK36* allele and carried the hypervirulence-associated *kfu* and yersiniabactin operons. Additionally, the isolate showed high survivability in the serum bactericidal and phagocytosis assays (82 and 94%, respectively) indicating that this is potentially a highly virulent clone.

In our study, the ST11, ST15, ST101, and ST258 isolates were detected in river sediment, river water, and wastewater. The presence of clinically relevant strains of *K. pneumoniae* in aquatic environments in association to human settlements indicates a potential for the environment as a transmission route of these strains. These isolates may have originated from human or animal guts colonized by the strains, and could have disseminated via feces and untreated wastewater. Although the data collected in this study is not sufficient to conclusively demonstrate transmission of *K. pneumoniae* strains between different environmental media, some interesting observations could nevertheless be made. For instance, KP8, isolated from pig manure, and KP11, isolated from vegetables, shared STs and were highly similar in the PFGE- and SNP-analysis, which may indicate the possibility of transmission from animals to humans via consumption of produce (Figure S1). The isolates were from the geographically separate villages C and I. Although of different STs, KP9, KP13, and KP14 also showed similar PFGE- and SNP-profiles, indicating that there may exist possible transmission routes between wastewater (KP14), river sediment (KP13), and soil (KP9). Bacteria in these environmental matrices could further potentially spread to humans, via for example surface and ground water, and irrigation of fields used for vegetable cultivation and finally consumption by humans. KP9, KP13, and KP14 were isolated from villages F, C, and D, respectively. Although villages C and D are located adjacent to each other, connected by a river, village F is separated from the aforementioned villages. Although the number of isolates in this study were too few to make a robust spatial analysis, the occurrence of genetically related isolates from different environmental matrices in geographically separate locations indicate the potential existence of a transmission mechanism.

## Conclusion

There is a scarcity of studies investigating occurrence of *K. pneumoniae* in the environment and the role of environmental transmission routes for dissemination of the pathogen. We sampled different environmental sources in a rural region of Shandong province, China, and screened the samples for ESBL-producing *K. pneumoniae*, and 6% of the samples were determined to be positive. All of the isolates were multidrug-resistant and several belonged to clinically relevant strains which are known to cause hospital outbreaks worldwide. Serotypes, virulence genes, serum survival, and phagocytosis survival were analyzed and showed the presence of virulence factors associated with highly virulent clones and a high degree of phagocytosis survivability, indicating the potential virulence of these isolates. The occurrence of ESBL-producing *K. pneumoniae* in the environment indicates inadequate treatment of domestic sewage and waste in the rural area. In addition, these results emphasize the need for further studies designed to elucidate the role of the environment in transmission and dissemination of ESBL-producing *K. pneumoniae* and the potential risk posed to human and environmental health.

## Ethics Statement

Ethical permission was granted by the Research Ethics Committee of the First Affiliated Hospital, College of Medicine, Zhejiang University, reference number 2018#1031.

## Author Contributions

XL, LN, CSL, JO, SB, BZ and BB conceived of and designed the study. XC, HZ, XJ and XL performed the sampling. XC and XJ performed the experiments. XC, BB and HZ analyzed the data. XC completed the first draft of the manuscript. BB, BZ, SB, JO, CSL, XL and LN critically revised the manuscript. All co-authors approved of the final version of the manuscript.

### Conflict of Interest Statement

The authors declare that the research was conducted in the absence of any commercial or financial relationships that could be construed as a potential conflict of interest.
