# Supplementary material for: Characterization of Clinically Relevant Strains of Extended-Spectrum β-Lactamase-Producing Klebsiella pneumoniae Occurring in Environmental Sources in a Rural Area of China by Using Whole-Genome Sequencing
Source: Front Microbiol. 2019 Feb 12;10:211. doi: 10.3389/fmicb.2019.00211 (PMC6379450; doi:10.3389/fmicb.2019.00211)
Supplement: Supplementary file 1 [file Table_1.docx]

Table S1. Whole-genome sequencing raw and assembly data for 14 isolates ESBL-producing *K. pneumoniae*.

| Sample | RawData | CleanPE | Ratio(%) | Asm_id | Kmer | Score | Number contigs | N50 | Len Longest contig | Totle_bases | Number contigs(>1k) | Totle bases contigs(>1k) |
| --- | --- | --- | --- | --- | --- | --- | --- | --- | --- | --- | --- | --- |
|  |  |  |  |  |  |  |  |  |  |  |  |  |
| KP1 | 1,435853564 | 1,362191838 | 94,8698 | 34 | 135 | 5442294 | 132 | 267340 | 1011218 | 5473672 | 54 | 5442294 |
| KP2 | 1,850816966 | 1,770933436 | 95,6838 | 35 | 137 | 5433610 | 187 | 207595 | 400279 | 5476180 | 80 | 5433610 |
| KP3 | 1,396136638 | 1,337670948 | 95,8123 | 31 | 129 | 5569466 | 154 | 350374 | 782714 | 5611850 | 55 | 5569466 |
| KP4 | 1,659582412 | 1,585856662 | 95,5575 | 33 | 133 | 5503496 | 150 | 232636 | 371892 | 5542933 | 60 | 5503496 |
| KP5 | 2,03001531 | 1,942401486 | 95,684 | 34 | 135 | 5404327 | 93 | 465704 | 1886679 | 5427636 | 28 | 5404327 |
| KP6 | 1,86197949 | 1,759831312 | 94,514 | 34 | 135 | 5441318 | 111 | 330561 | 442773 | 5467731 | 44 | 5441318 |
| KP7 | 1,643578224 | 1,551017338 | 94,3683 | 33 | 133 | 5325462 | 133 | 279815 | 582542 | 5349520 | 66 | 5325462 |
| KP8 | 1,75556828 | 1,668801566 | 95,0576 | 34 | 135 | 5383987 | 120 | 254137 | 930466 | 5408625 | 53 | 5383987 |
| KP9 | 1,952960312 | 1,867384686 | 95,6181 | 35 | 137 | 5378353 | 133 | 232556 | 412457 | 5406297 | 60 | 5378535 |
| KP10 | 2,479562242 | 2,295476028 | 92,5758 | 36 | 139 | 5581395 | 127 | 242437 | 518772 | 5608537 | 49 | 5581395 |
| KP11 | 2,060746226 | 1,866134406 | 90,5562 | 35 | 137 | 5383147 | 116 | 240874 | 471249 | 5405498 | 53 | 5383147 |
| KP12 | 1,649473868 | 1,556040504 | 94,3355 | 34 | 135 | 5277254 | 153 | 305143 | 767911 | 5323806 | 50 | 5277254 |
| KP13 | 2,036193022 | 1,927728816 | 94,6731 | 34 | 135 | 5476684 | 99 | 252274 | 551353 | 5493155 | 55 | 5476684 |
| KP14 | 0,940537324 | 0,893949898 | 95,0467 | 29 | 125 | 5797385 | 247 | 210292 | 632959 | 5855036 | 106 | 5797385 |
