# Supplementary material for: Characterization of Clinically Relevant Strains of Extended-Spectrum β-Lactamase-Producing Klebsiella pneumoniae Occurring in Environmental Sources in a Rural Area of China by Using Whole-Genome Sequencing
Source: Front Microbiol. 2019 Feb 12;10:211. doi: 10.3389/fmicb.2019.00211 (PMC6379450; doi:10.3389/fmicb.2019.00211)
Supplement: Supplementary file 2 [file Table_2.DOCX]

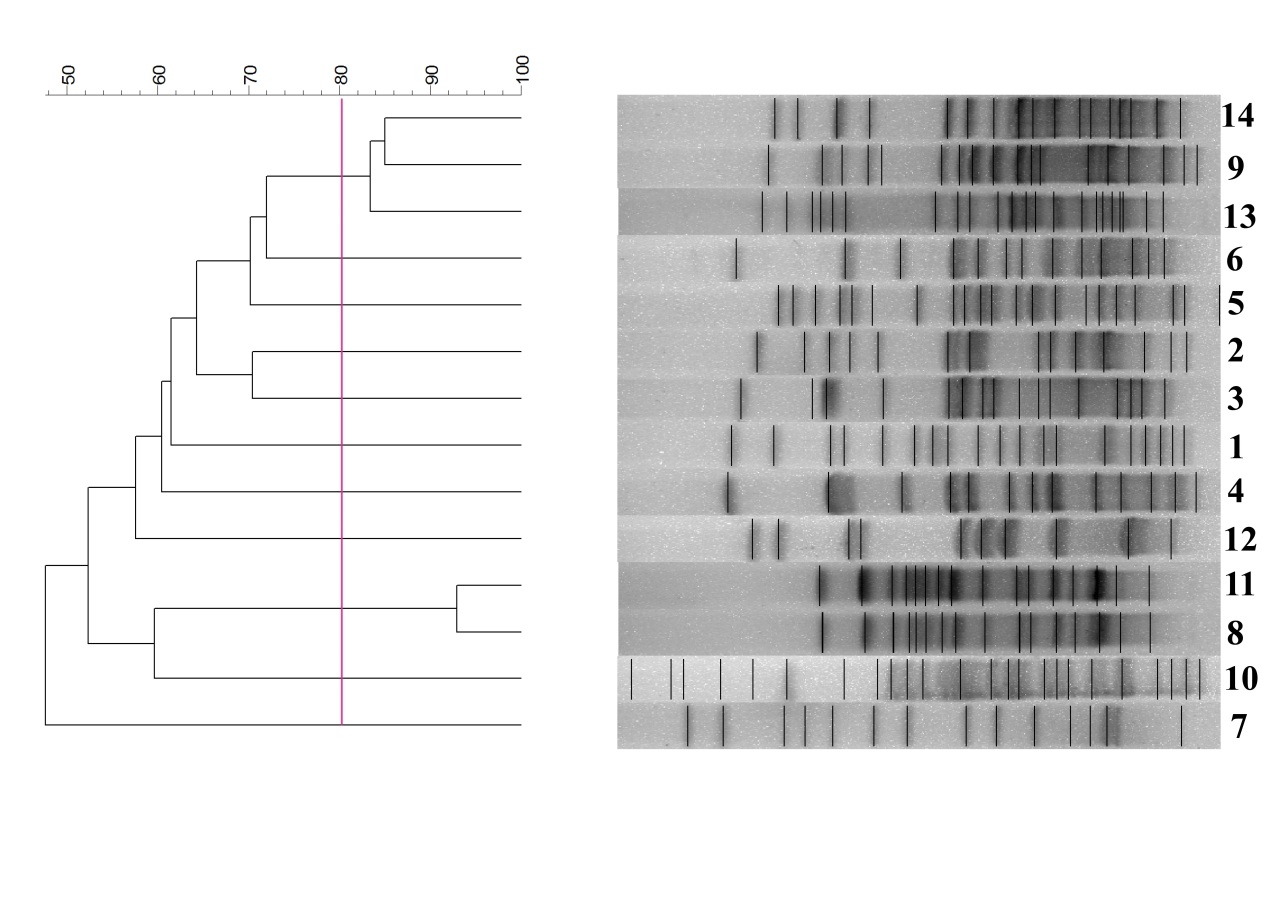


**Fig. S1.** Dendrogram of ESBL-producing *K. pneumoniae* based on PFGE with XbaI-digestion. The blue line indicates the 80%-similarity cut-off used for determining genetic relationship.
